# Supplementary material for: The Effect of Sadness on Visual Artistic Creativity in Non-Artists
Source: Brain Sci. 2023 Jan 15;13(1):149. doi: 10.3390/brainsci13010149 (PMC9856421; doi:10.3390/brainsci13010149)
Supplement: Supplementary file 1 [file brainsci-13-00149-s001.zip › brainsci-2122190-SI.pdf]

Supplementary materials

10 pieces of music per condition self-selected by participants (singer-title)

Positive condition – Happy music

1. ABBA – Gimme! Gimme! Gimme! (A man after midnight)
2. Simple Minds – See the lights
3. Bob Sinclair and Raffaella Carrà – A far l'amor comincia tu
4. Depeche Mode – Personal Jesus
5. Simone Cristicchi – Vorrei cantare come Biagio
6. Michael Jackson – Billy Jean
7. Bruno Mars – The Lazy song
8. Grace Jones – I have seen that face before
9. Michael Sembello – Maniac
10. LMFAO – Sexy and I know it

Negative condition – Sad music

1. James Blunt – Goodbye my lover
2. Ennio Morricone – Strana bambina
3. Depeche Mode – Waiting for the night
4. Lucio Battisti - Emozioni
5. Fabrizio De André – La canzone dell'amore perduto
6. Yiruma – River flows in you
7. Yumeji's Theme – In the mood for love
8. Bruce Springsteen - Philadelphia
9. Yann Tiersen – Good bye Lenin
10. Distrubed – The sound of silence

Neutral condition

1. Jovanotti – A te
2. Ligabue – Una vita da mediano
3. Annalisa – Dieci
4. Fedez fit Francesca Michelino – Magnifico
5. George Michael – Careless Whisper
6. Gustave Holst – The planets
7. Cesare Cremonini – Marmellata #25
8. Tatanai – Quelli come noi
9. Lemandorle – Ti amo il venerdì sera
10. Le Donatella – Scarpe Diem
